# Class II two-peptide lanthipeptide proteases: exploring LicTP for biotechnological applications

**DOI:** 10.1007/s00253-023-12388-5

**Published:** 2023-02-10

**Authors:** Joana C. Barbosa, Eva Mösker, Raquel Faria, Roderich D. Süssmuth, Sónia Mendo, Tânia Caetano

**Affiliations:** 1 Department of Biology and Centro de Estudos Do Ambiente E Do Mar (CESAM), Universidade de Aveiro, Aveiro, Portugal; 2 Centro de Biotecnologia E Química Fina, Escola Superior de Biotecnologia, Universidade Católica Portuguesa, Centro Regional Do Porto, Porto, Portugal; 3 Institut Für Chemie, Technische Universität Berlin, Berlin, Germany

**Keywords:** Lanthipeptides, Lantibiotics, Site-directed mutagenesis, Chimeric genes, Recombinant peptides, Proteases

## Abstract

**Abstract:**

The enzymatic machinery involved in the biosynthesis of lantibiotic is an untapped source of proteases with different specificities. Lanthipeptide biosynthesis requires proteolysis of specific target sequences by known proteases, which are encoded by contiguous genes. Herein, the activity of lichenicidin A2 (LicA2) trimming proteases (LicP and LicT) was investigated in vivo. Firstly, the impact of some residues and the size of the peptide were evaluated. Then followed trials in which LicA2 leader was evaluated as a tag to direct production and secretion of other relevant peptides. Our results show that a negatively charged residue (preferably Glu) at cleavage site is important for LicP efficacy. Some mutations of the lichenicidin hexapeptide such as Val-4Ala, Asp-5Ala, Asn-6Ser, and the alteration of GG-motif to GA resulted in higher processing rates, indicating the possibility of improved lichenicidin production in *Escherichia coli*. More importantly, insulin A, amylin (non-lanthipeptides), and epidermin were produced and secreted to *E. coli* supernatant, when fused to the LicA2 leader peptide. This work aids in clarifying the activity of lantibiotic-related transporters and proteases and to evaluate their possible application in industrial processes of relevant compounds, taking advantage of the potential of microorganisms as biofactories.

**Key points:**

• *LicM2 correct activity implies a negatively charged residue at position -1*.

• *Hexapeptide mutations can increase the amount of fully processed Bliβ*.

• *LicA2 leader peptide directs LicTP cleavage and secretion of other peptides*.

**Graphical abstract:**

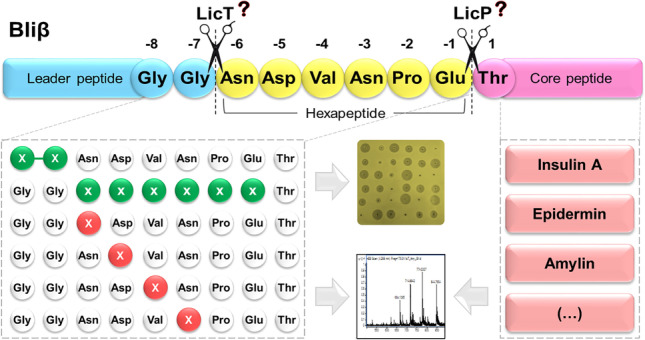

**Supplementary Information:**

The online version contains supplementary material available at 10.1007/s00253-023-12388-5.

## Introduction

Lantibiotics are lanthipeptides with antimicrobial activity that have been the subject of intense research due to their bioactivity against clinically relevant strains having low toxicity towards mammals (van Heel et al. 2011; Dischinger et al. 2014; Barbosa et al. 2015, 2022b; Field et al. 2015; Basi-Chipalu 2016). During their processing, the installation of the characteristic lanthionine (Lan) and methyllanthionine (MeLan) rings is catalysed by dedicated modifying enzymes, which vary in architecture and mechanism depending on the lanthipeptide class. All lantibiotics isolated so far belong either to class I or class II. In these two classes, after the formation of Lan/MeLan by LanBC or LanM enzymes, respectively, the modified core peptide is transported to the extracellular environment and the N-terminal region (leader peptide) is removed (Arnison et al. 2013; Barbosa et al. 2015; Repka et al. 2017; van Staden et al. 2021). In class II lantibiotics, LanTp transporters promote the export of the active lantibiotic. In addition, they have a proteolysis domain which makes them bifunctional molecules that cleave the lantibiotic leader peptide after the GlyGly-motif (GlyGly/GlyAla/GlySer) (Barbosa et al. 2015; Tang et al. 2015). Class II includes two-peptide lantibiotics, in which two different peptides (α and β) act synergistically to exhibit antibacterial activity. Examples include lichenicidin (Bliα and Bliβ) and haloduracin (Halα and Halβ), among others (Lawton et al. 2007; Oman and van der Donk 2009; Shenkarev et al. 2010; Barbosa et al. 2022a). The precursor peptides (LanA1 and LanA2) are modified by two dedicated LanM enzymes, but the removal of their respective leader peptides and their export are promoted by the same LanTp. In β-peptides, it is common that a short oligopeptide tag remains attached to the N-terminal and is then trimmed by a subtilisin-like extracellular serine protease (LanP) (Caetano et al. 2011; Wang et al. 2014). This tag is generally composed of 6 amino acids and is referred to as hexapeptide. LanP enzymes can be encoded in the lantibiotic biosynthetic cluster, as it is the case of lichenicidin, or may be of unknown origin, as with haloduracin (Cooper et al. 2008; Dischinger et al. 2009; Shenkarev et al. 2010). The in vitro activity of lichenicidin LanP (LicP) was investigated and allowed to establish that the trimming of LicP i) occurs in the presence of the leader peptide (prior cleavage by LicT not required), ii) occurs with or without the installation of Lan/MeLan modifications, and iii) is dependent on the Glu-1 residue of the hexapeptide (Tang et al. 2015). Apart from being recognized by LanP, the biological role of the hexapeptide remains unclear. It has been suggested that it can be a recognition tag of the posttranslational processing enzymes, since it is part of the leader peptide. In addition, it may contribute to the immunity of the producer strain, since its presence keeps the β-peptide inactive until it reaches the extracellular environment, where it can interact with the α-peptide. Thus, lantibiotic producers are also natural sources of new proteases with novel specificities and, possibly, several stability-related features (Tang et al. 2015). In addition, they are sources of different secretion systems directed by specific leader peptides. As such, these systems can be exploited to develop novel “in vivo biocatalytic factories” involving microorganisms capable of recombinant DNA technology, such as *Escherichia coli*. For example, the substitution of the Halβ hexapeptide for Bliβ together with LicP allowed the development of a colony-based approach with potential application in the screening of ribosomally synthetized and post-translationally modified peptides analogues in this Gram-negative heterologous host (Si et al. 2018).

**Fig. 1:**
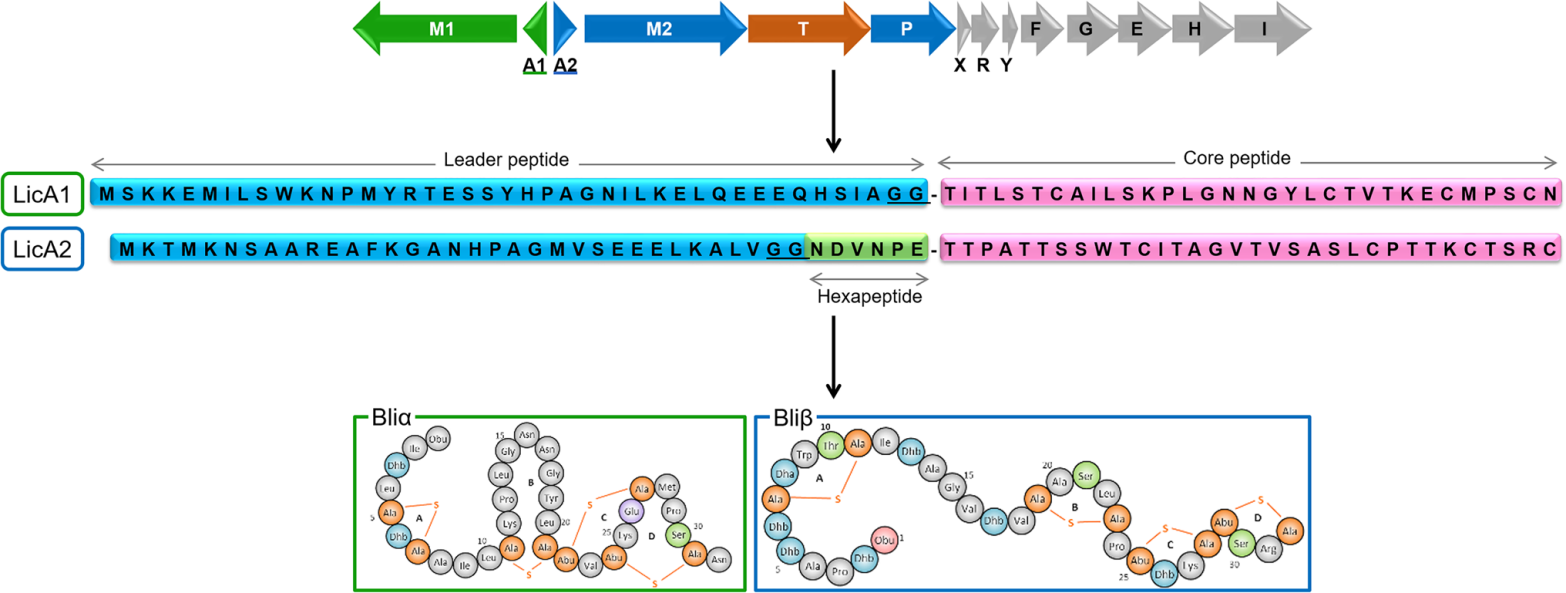
Lichenicidin gene cluster containing the genes essential for the production of Bliα (green) and for the production of Bliβ (blue); LicT is responsible for cleaving the precursor peptides C-terminally of the double-Gly motif (underlined) and for the transport of both peptides out of the cell. LicP cleaves the remaining hexapeptide attached to Bliβ in the extracellular space; in gray are other genes encoding putative regulatory enzymes and self-immunity genes, not essential for lichenicidin production in *E. coli*. Adapted from Caetano et al. (2011)

Here, we applied a fully in vivo approach to investigate the activities of LanP and LanT involved in the biosynthesis of two-peptide lantibiotics using the system developed for the heterologous expression of lichenicidin, which was the first lantibiotic to be produced fully in vivo in *E. coli* (Caetano et al. 2011). Specifically, the study was conducted with enzymes LicT and LicP of lichenicidin and the Bliβ peptide (Fig. 1). Also, we tested the application of the Bliβ leader as a tag to promote secretion of other lantibiotics or peptides with medical application, namely insulin A, amylin, and lunasin.

## Material and methods

### Construction and analysis of the hexapeptide mutants

**Fig. 2:**
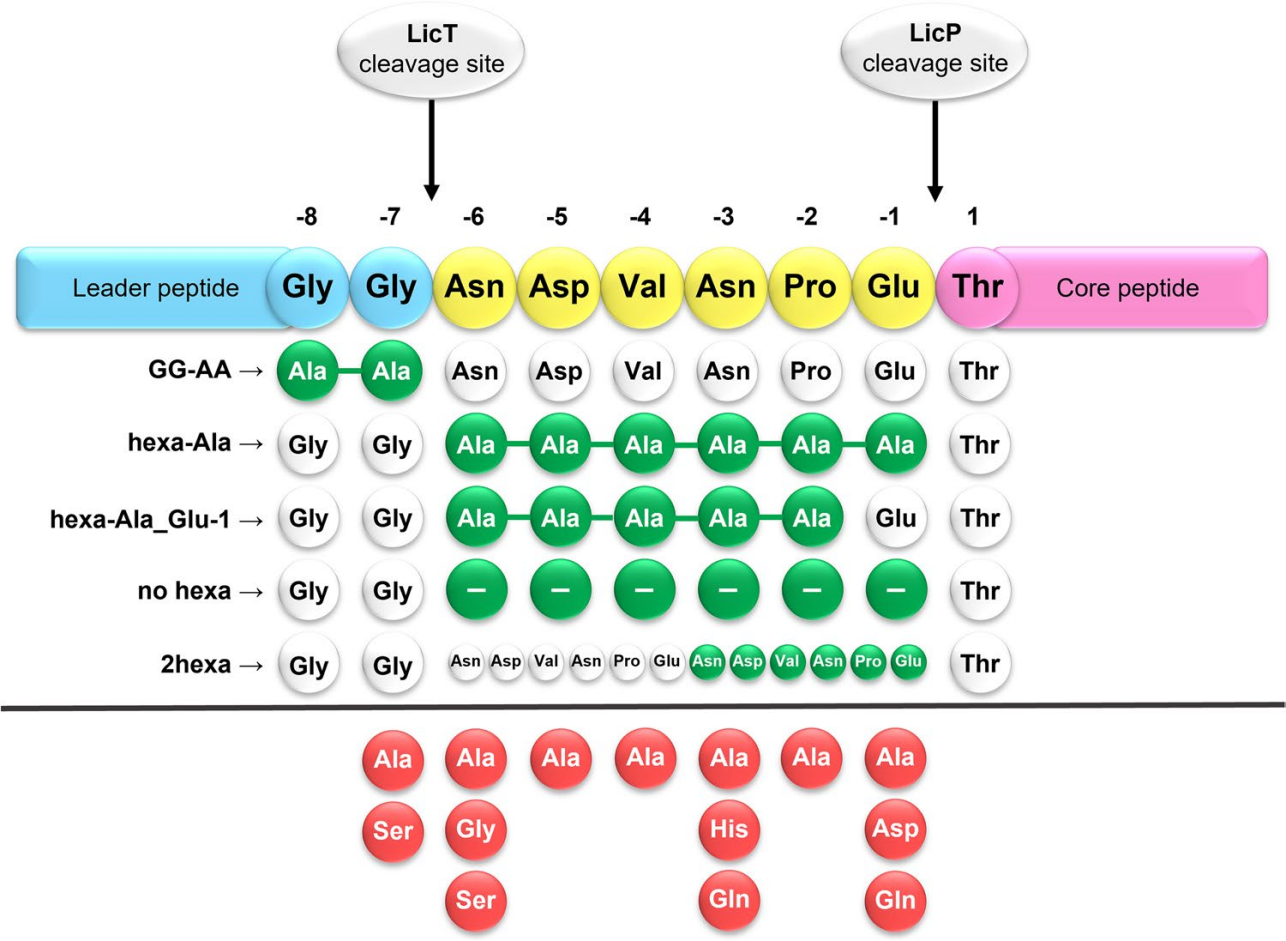
Schematic representation of the mutations inserted into Bliβ’s hexapeptide. The native sequence is shown on top: hexapeptide in yellow, core peptide in pink, and the remaining leader sequence in blue. The mutations inserted are represented in green when multiple amino acids were replaced or in red for single amino acid mutations. Deletion of an amino acid is represented by a circle containing a “–.” The cleavage sites of LicT and LicP enzymes are also represented

Hexapeptide and the GlyGly-motif mutations were performed by site directed mutagenesis (SDM) of the *licA2* gene cloned into the pET24a + vector (Fig. 2) as previously described by Barbosa et al. (2019). A total of 18 hexapeptide and 3 double Gly-motif mutants were obtained. Specific primers for each mutation were designed using the web-based PrimerX tool and are listed in Table S1. *E. coli* BL21(DE3)Gold containing a fosmid encoding the entire lic gene cluster, except the structural genes, was transformed with the plasmid containing each of the desired mutations. The host was allowed to grow and the supernatant was extracted with 1-butanol, as previously described by Barbosa et al. (2019)*.* Extracts were then suspended in 100 µL 70% ACN and 15 µL was applied for HPLC–MS analysis, using multiple reaction monitoring (MRM) mode on an ESI-Triple-Quadrupole-MS 6460 series (Agilent Technologies, Germany) coupled to an Agilent 1290 Infinity HPLC system (Agilent Technologies, Germany). Separation was performed with a Poroshell 120 EC-C8 (2.1 × 50 mm, 2.1 µm) column with a precolumn Poroshell 120 EC-C8 (2.1 × 5 mm, 2.1 µm) and with the following gradient: from 5 to 20% of solvent B over 0.5 min, increased to 70% of B over 4.5 min, followed by 100% B over 6 min with a flowrate of 0.5 mL/min. The solvent A was H_2_O with 0.1% formic acid and solvent B was acetonitrile with 0.1% formic acid. For quantification of lichenicidin peptides, [M + 3H]^3+^ adducts were used as precursor ions. For MRM, mass transitions for Bliβ m/z 1007.8 → 1302.0 and m/z 1007.8 → 264.9 were used and compared with a standard calibration curve obtained with pure Bliα and Bliβ (Fig. S1). When required, an MS2Scan of the sample was also performed for identification of masses, other than the native peptides. The bioactivity of all the extracts was assessed by deferred antagonistic bioassay against the Gram-positive indicator strain *Kocuria rhizophila* ATCC 9341, where the complementary peptide required for synergistic bioactivity was incorporated in the bioassay plate as described by Barbosa et al. (2019). All the experiments were performed in triplicate and the statistical analyses of bioactivity and quantification were performed with one-way ANOVAs. Whenever the data failed to meet the normality, one-way ANOVAs on ranks were used. The resulting data was analysed with SigmaPlot 11.0 and the significance defined was *p* < 0.05 for all the analyses.

### Construction of the recombinant genes

The recombinant genes containing the leader sequence of the *licA2* gene-fused upstream of the peptide of interest were synthesized by General Biosystems (USA). Each recombinant gene was amplified, digested with *Nde*I and *Xho*I, and cloned into pET-24a vector (containing a kanamycin resistance cassette). *licTP* amplicon was amplified from pHPβ vector, developed by Kuthning et al. (2015), which contains an optimized promotor and an ATG starter codon in the *licP* gene. The amplicons were digested with *Sal*I and *Nco*I and cloned into pCDFDuet-1 vector (streptomycin resistance cassette). All the primers used in this study are listed in Table S2. *E. coli* BL21(DE3)Gold chemically competent cells were transformed with two plasmids: one containing the recombinant gene and the other encoding the enzymes LicT and LicP. *E. coli* expressing only the recombinant gene was used as control for each condition. The expected peptides and respective masses are listed in Table 1. All the recombinant genes were cloned in frame with a C-terminal His_6_-Tag, to facilitate purification, regardless of the proteolytic cleavage the peptide may have undergone.

**Table 1:** List of peptides used in this study and their respective masses

Peptide	Origin	Amino acid sequence	Peptide mass (Da)	Applications	Reference
Insulin A	Human	GIVEQCCTSlCSLYQLENYCN	2485	Diabetes therapeutics	(Göddel et al. 1979; Miller and Baxter 1980; Zündorf and Dingermann 2001)
Epidermin	*Staphylococcus epidermidis*	IASKFICTPGCAKTGSFNSYCC	2164	Antimicrobial agent (lantibiotic)	(Allgaier et al. 1985; Kupke et al. 1993)
Amylin	Human	KCNTATCATQRLANFLVHSSNNFGAILSSTNVGSNTY	4005	Inhibits secretion of growth hormone	(Krampert et al. 2000; Lopes et al. 2004; Lutz and Meyer 2015; Mazor et al. 2002; Yonemoto et al. 2009)
Plantaricin E	*Lactobacillus plantarum*	FNRGGYNFGKSVRHVVDAIGSVAGIRGILKSIR	3644	Antimicrobial agent (bacteriocin, not lantibiotic)	(Diep and Nes 1996; Pal and Srivastava 2014, 2015)
Lunasin	Soy, barley, wheat	SKWQHQQDSCRKQLQGVNLTPCEKHIMQKIQRGDDDDDDDDD	5125	Chemopreventive peptide	(Kyle et al. 2012)
Somatostatin-14	Human	AGCKNFFWKTFTSC	1639	Prevention of aging-associated diseases, including Alzheimer’s disease and type II diabetes	(Itakura et al. 1977; Maicas et al. 2013)
Mersacidin	*Bacillus* spp.	CTFTLPGGGGVCTLTSECIC	1825	Antimicrobial agent (lantibiotic)	(Niu and Neu 1991; Bierbaum et al. 1995)

### Recombinant peptide extraction using Ni–NTA affinity beads

Recombinant peptides were extracted with Ni–NTA beads (Qiagen, Germany) due to their high affinity to the C-terminal His_6_-Tag. *E. coli* cultures expressing the recombinant genes were grown overnight, at 37 °C and 180 rpm, in a final volume of 3 mL Luria–Bertani broth (LB) containing the appropriate selective markers. From this overnight culture, 1 mL was used to inoculate 100 mL of fresh LB and the cells were allowed to grow for 24 h under the same conditions. Cells were harvested by centrifugation at 4 °C, 3220 × g for 20 min. The supernatants were kept on ice until extraction with the Ni-beads. The cellular pellets were weighed and dissolved in 10 mL per 1 g of cells using binding buffer (50 mM NaH_2_PO_4_, 300 mM NaCl, 20 mM imidazole, adjusted to pH 8.0 with NaOH solution) containing DNase I (5 µg/mL). The cells were lysed with 10 mg/mL of lysozyme and sonicated for 15 min on ice. Then, lysates were incubated on ice for 15 min and centrifuged at 4 °C, 10,000 × g for 1 h. The intracellular, soluble fraction was collected and placed on ice. The adequate amount of Ni–NTA resin was washed and equilibrated with the binding buffer, according to the manufacturer’s instructions, and added to the supernatants and to the intracellular soluble fractions. The mixture was incubated at 4 °C for 4 h with gentle shaking, and then centrifuged at 3220 × g at 4 °C for 1 min to collect the Ni beads. The beads were washed twice with 10 mL of binding buffer, centrifuged as mentioned above, and the supernatant was discarded. After washing, the beads were transferred to a new centrifuge tube, using 1 mL of binding buffer. After centrifugation for 1 min at 4 °C, 6000 × g, the supernatant was discarded and the peptides were eluted with elution buffer (50 mM NaH_2_PO_4_, 300 mM NaCl, 250 mM imidazole, adjusted to pH 8.0 with NaOH solution). The elution step was repeated 5 times, collecting all the supernatant for further analysis.

### Analytics of recombinant peptides

Before analysis, samples were desalted to remove excess of salts using the drop dialysis technique, with cellulose-ester membrane discs (Millipore, Germany). A 10 µL aliquot of the desalted samples was analysed in a 6530 Accurate Mass Q-ToF (Agilent Technologies, Germany) coupled to an Agilent 1260Infinity HPLC system (Agilent Technologies). Separation was performed with a C5 Supelco Bio Wide Pore column (100 × 2.1 mm, 5 µm) using the following gradient: from 5 to 100% of solvent B over 10 min, followed by 1 min at 100% of B, and decreasing again to 5%, with a flow rate of 0.5 mL/min. The following solvents were used: H_2_O with 0.1% formic acid (solvent A) and acetonitrile with 0.1% formic acid (solvent B). The Q-ToF spectrometer is equipped with a Dual AJS ESI Source and was set to the following parameters: gas temperature 300 °C, drying gas 8 L/min, sheath gas 350 °C, capillary voltage 3500 V, and nozzle voltage 0 V. The samples were run in scan mode and the predicted molecular masses were identified (Table S3).

## Results

### The hexapeptide of Bliβ and its putative role in in vivo biosynthesis and activity

**Fig. 3:**
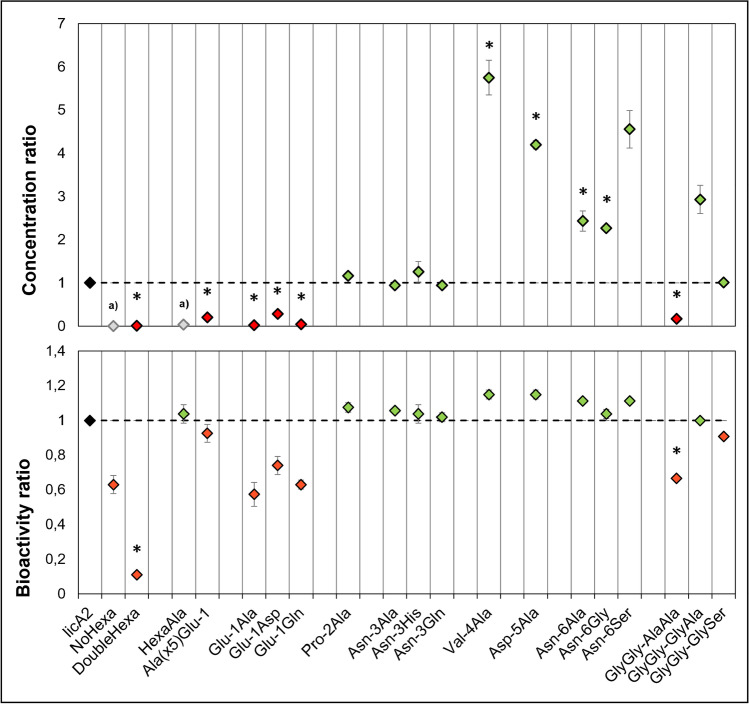
Quantification of peptide (top) and bioactivity against *Kocuria rhizophila* (bottom) of Bliβ hexapeptide mutants. The ratio between the native Bliβ concentration/activity (LicA2) is represented in black, green diamonds represent increased quantity/activity, and red diamonds decreased. * indicates statistically significant differences in bioactivity and quantification compared to the control (*p* < 0.05). ^a)^ Fully modified Bliβ not detected

To better understand whether the hexapeptide is required for Bliβ production in vivo, two mutants were constructed: one without the hexapeptide (NoHexa, Fig. 2) and another where its residues were replaced by Ala (HexaAla, Fig. 2). In the *E. coli* extracts producing the NoHexa and the HexaAla variants, the mature Bliβ was not detected by HPLC–ESI–MS, not even when using multiple reaction monitoring (MRM) mode, which is a significantly more sensitive method. The extracts retained 50% (NoHexa) or even full (HexaAla) activity (Fig. 3), which implies the presence of a Bliβ variant. MS2scan method identified masses corresponding to incomplete dehydrated Bliβ variants, not detected in the control extracts (masses corresponding to + 5 dH_2_O and + 1dH_2_O in the NoHexa and HexaAla extracts, respectively; Fig. S2). The exact nature and position of these changes have not yet been analysed, as the variants were recovered in low amounts, and are therefore insufficient for further studies. However, we concluded that the dehydration stage of Bliβ was altered in both mutants, although at a great degree if no hexapeptide is present. These results suggest that the presence of a hexapeptide after the GlyGly-motif is relevant for LicM2 functionality. We also tested the duplication of the hexapeptide (DoubleHexa mutant; Fig. 2) that resulted in the production of fully dehydrated Bliβ, albeit in lower amounts that justifies the low bioactivity of the extract (Fig. 3).

### Significance of LicP proteolysis site alteration in the Bliβ production

**Fig. 4:**
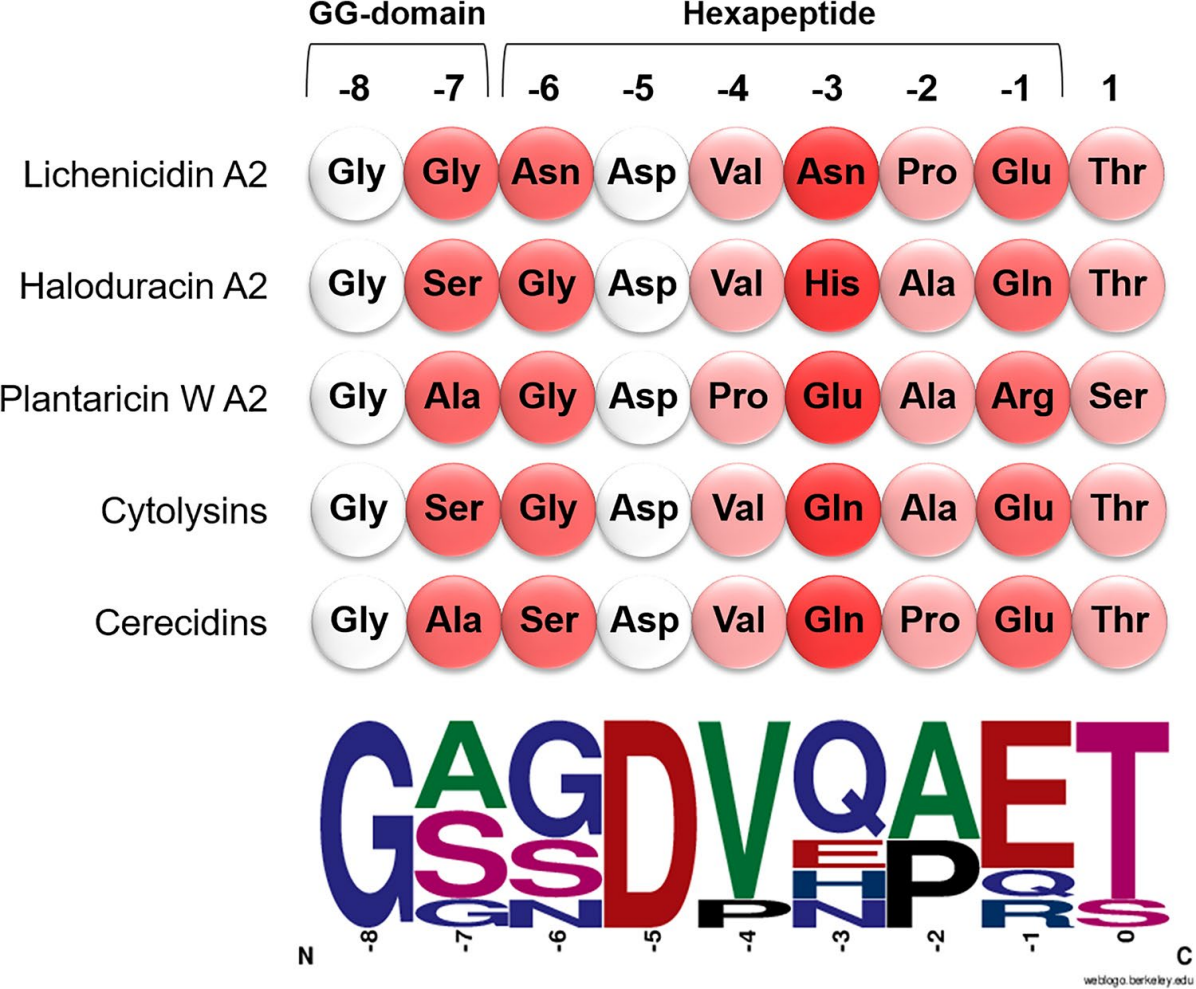
Alignment of LicA2 with hexapeptides from other closely related lantibiotics: haloduracin A2, plantaricin W A2, cytolysins, and cerecidins. White circles represent amino acids conserved among all the sequences, while the decrease in the conservation level is represented by red circles: light red for higher conservation and dark red for lower. At the bottom, representation of the relative frequency of hexapeptide amino acids

Another objective was to understand which hexapeptide’s amino acids are essential for LicP trimming in vivo, and to that end we performed an Ala-scanning of its six residues (Fig. 2). Glu-1 is predicted to be particularly important since it is the LicP cleavage site (Fig. 1). The *E. coli* expressing Glu-1Ala was the only one in which Bliβ production decreased significantly and was also accompanied by a reduction in bioactivity (Fig. 3). Similar results were obtained when Glu-1 was replaced by the isosteric but uncharged Gln, or by Asp, which maintains the negative charge at this position. Gln is found at position -1 of other hexapeptides, like haloduracin A2 (HalA2; Fig. 4). Results obtained by replacing Glu-1Ala raised doubts on the results of HexaAla: the lack Bliβ identification was mostly due to Glu-1Ala substitution? This would imply that in HexaAla mutant extracts, fully dehydrated Bliβ was present, but it was not detected due to deficiency in Ala hexapeptide removal. To answer this question, a new mutant was generated in which all the residues of the hexapeptide were replaced by Ala, except Glu-1(Ala_(x5)_Glu-1). Comparison of HexaAla and Ala_(x5)_Glu-1 extracts showed that the latter produced fully dehydrated Bliβ, and slight, not significant, decrease in bioactivity was observed (Fig. 3). This result supports the fact that the presence of the hexapeptide, even composed by different residues, is essential for the core peptide correct dehydration. For the other single mutations in the hexapeptide, it was observed that the replacement of the residues -4, -5, and -6 by Ala led to a significant increase in Bliβ yields (Fig. 3). Substitutions at hexapeptide’s intermediate positions, -2 and -3, had no impact on Bliβ production and bioactivity (Fig. 3). Pro-2Ala did not affect Bliβ production, which is consistent with the fact that this residue is not essential for LicP recognition of the LicA2 hexapeptide in vitro (Tang et al. 2015). Other lantibiotics have either Pro or Ala at this position (Fig. 4). The residues -3 and -6 are the least conserved amino acids in the hexapeptides of lichenicidin’s closely related lantibiotics (Fig. 4). For this reason, we replaced Asn-3 by His and Gln, and Asn-6 by Gly and Ser. Results show that no significant differences in bioactivity or Bliβ abundance were observed for Val-3His and Val-3Gln mutants (Fig. 3). Regarding the extracts of Asn-6 derivatives, these showed significantly higher yields of Bliβ (approximately 3 times higher), when compared with the wild-type extracts (*p* < 0.05; Fig. 3).

### Interplay between LicT and LicP proteolysis

Before LicP proteolysis at the extracellular space, the maturation of the precursor peptide LicA2 into Bliβ requires the removal of the leader peptide by LicT at the GlyGly-motif, simultaneously with the secretion process. It is known that alteration of the GlyGly-motif to AlaAla prevents the removal of the leader (Uguen et al. 2005). So, as expectable, this mutation caused a significant decrease in the abundance of Bliβ, but it was still identified by HPLC–MS (Fig. 3). This result shows that the modified peptide with the leader peptide attached can still be a substrate for LicP, although with a negative impact in its yield. In class II lanthipeptides, natural variants of the GlyGly-motif exist that include GlyAla and GlySer (Fig. 4). We tested these variants and observed that Bliβ abundance was significantly increased when the GlyGly was changed to GlyAla and remained unaltered when changed to GlySer (Fig. 3).

### Assessment of LicA2 leader peptide and LicTP as a replacement for the secretion system of other core peptides

**Fig. 5:**
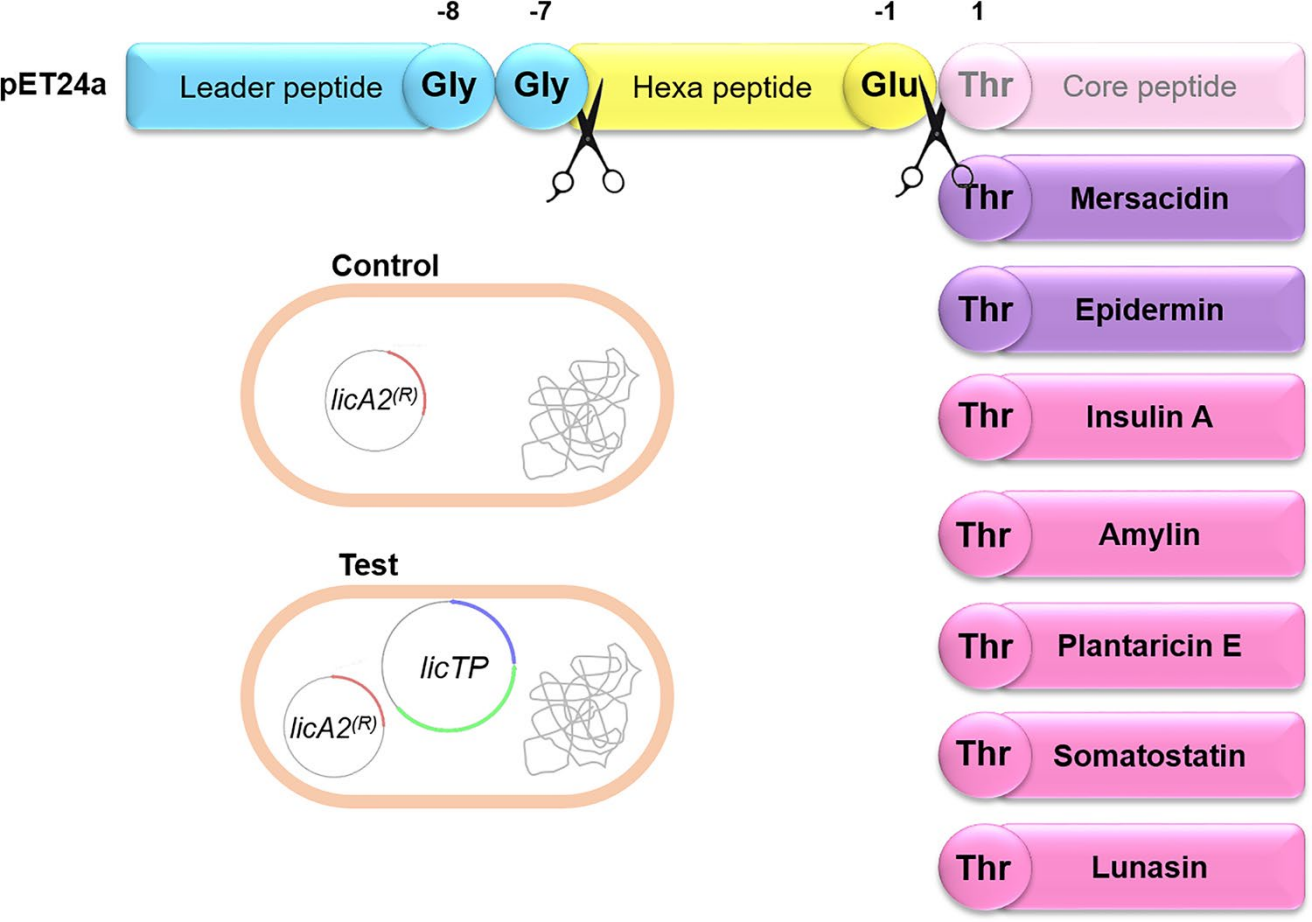
Representation of constructions made with chimeric genes. In blue, LicA2 leader sequence with double Gly motif highlighted; in yellow, the hexapeptide with a Glu residue in position -1; in pink, the core peptide is indicated, which is replaced by various core sequences; position 1 was mutated to Thr if required to maintain the cleavage site. Conditions tested: control, LicP cleavage, LicT cleavage, and transport; LicTP combined activity

Another aspect that we wanted to clarify was whether LicA2 leader peptide, together with LicT and LicP, could function as a secretion system of other peptides in *E. coli*. As a proof of concept, seven peptides (insulin A, epidermin, amylin, plantaricin E, lunasin, somatostain-14, and mersacidin) were selected in view to their potential medical or industrial applications and to test non-lanthipeptides. All the peptides, except mersacidin, were previously reported to be expressed in *E. coli* (Table 1 and Fig. 5). They were fused to LicA2 leader peptide and expressed with or without LicTP. Their presence was screened by HPLC–ESI–MS inside the soluble cellular fraction and in the supernatant. Of the seven peptides tested, epidermin and mersacidin are class I and class II lanthipeptides, respectively, and were expected to be linear and non-dehydrated because their dedicated LanM enzymes were not included in the expression system. Results showed insulin A, amylin, and epidermin in the supernatant without LicA2 leader peptide attached (with LicTP), but not in the control system (without LicTP; Tables 2, S3, and S4). Therefore, LicTP were able to secrete and cleave these three peptides. Plantaricin E was detected only in soluble cellular fraction (Tables 2, S3, and S4). Considering lunasin, only the peptide fused to LicA2 leader peptide was detected and in both cellular and supernatant fractions (Tables 2 and S3). Lastly, somatostatin-14 and mersacidin were not detected either inside or outside the cells, not even their possible fused forms (Table 2).

**Table 2:** Peptides or their derivatives identified in the soluble cellular fractions and in the supernatant of *E. coli* in the presence (+ LicTP) or absence of LicTP (− LicTP). ND indicates that none of the peptides was detected

Peptide	Variant	+ LicTP		− LicTP (control)	
		Soluble cellular fraction	Supernatant	Soluble cellular fraction	Supernatant
Insulin A (InsA)	Leader + hexa + peptide	** + **	** + **	** + **	** + **
	Hexa + peptide	** + **	** + **	−	−
	Peptide	** + **	** + **	−	−
Epidermin (Epi)	Leader + hexa + peptide	** + **	** + **	** + **	** + **
	Hexa + peptide	−	** + **	−	−
	Peptide	−	** + **	−	−
Amylin (Amy)	Leader + hexa + peptide	** + **	** + **	−	−
	Hexa + peptide	−	−	−	−
	Peptide	** + **	** + **	−	−
Plantaricin E (PlnE)	Leader + hexa + peptide	** + **	−	+	−
	Hexa + peptide	** + **	−	−	−
	Peptide	** + **	−	−	−
Lunasin (Lun)	Leader + hexa + peptide	** + **	** + **	** + **	** + **
	Hexa + peptide	−	−	−	−
	Peptide	−	−	−	−
Somatostatin-14	All	ND	ND	ND	ND
Mersacidin	All	ND	ND	ND	ND

## Discussion

Together, our results suggest that the hexapeptide might have an additional biological role, in addition to being a recognition site for LicP, since it seems to be determinant for dehydration of LicA2 core peptide by LicM2. One hypothesis that deserves further investigation is that hexapeptides might help LicM enzymes to distinguish and select their cognate peptides. Regarding the relevance of Glu-1 residue in the hexapeptide, we concluded that Glu-1Asp is better tolerated than Glu-1Ala or Glu-1Gln mutations (Fig. 3), suggesting that a negative charge is important for the removal of the hexapeptide by LicP. This is in accordance with in vitro studies of Tang et al. (2015) that established some interactions between Glu-1 and specific residues from the catalytic site of LicP. Thus, the presence of a Glu residue at position -1 ensures the production of mature and fully dehydrated Bliβ. The outcome of Ala scanning of the remaining residues of the hexapeptide showed that -4, -5, and -6 substitutions by Ala improved Bliβ yields. Thus, in vivo, LicP tolerates changes in the hexapeptide region structurally proximal to its active site. This was unexpected for Val-4 and Asp-5, because in vitro assays showed that Ala substitutions of these two residues decrease the efficiency of cleavage (Tang et al. 2015). More specifically, Val-4 appears to interact with a pocket within LicP structure where only small hydrophobic amino acids fit, as it is the case of both Val and Ala (Tang et al. 2015). On the other hand, the Val-4Ala mutation makes the hexapeptide more similar to the LicP linker sequence (Asn-Thr-**Ala**-Val-Asn-Glu) found between its pro- and catalytic domains. This linker is cleaved in an autocatalytic process to yield the active catalytic domain of LicP (Tang et al. 2015). Additionally, we determined that Asn-6Ser substitution was particularly beneficial in terms of peptide yield, as the Bliβ abundance was approximately 3 times higher than that of the wild type. This position seems to be highly permissive, and its manipulation might contribute to increase Bliβ production, all well as other peptides, in *E. coli*.

Regarding LicT and LicP functions, we confirmed that LicP is also capable of removing the leader peptide attached to the hexapeptide in vivo, when proteolysis by LicT is impaired, as suggested previously by in vitro experiments (Tang et al. 2015). Nonetheless, owing to the lower abundance of Bliβ in the extracts with disrupted GlyGly-motif, the reaction is favoured if preceded by LicT proteolysis. LicT proteolysis domain recognizes all the three GlyGly-motifs described so far for class II lanthipeptides (GlyGly, GlySer, and GlyAla), but in *E. coli*, the Bliβ yields are higher with the GlyAla-motif. This is not universal, since GlyGly → GlyAla substitution in the mutacin precursor peptide caused the accumulation of dehydrated premutacin in the cell membrane (Chen et al. 2001). The lacticin 481 GlyGly-motif was intensively mutated and the only mutation tolerated was GlyAla → GlyGly, resembling the straight double Gly-motif (Furgerson Ihnken et al. 2008).

The heterologous expression and secretion of add-value peptides in biofactories such as *E. coli* are desirable since it can facilitate their purification and, consequently, ease downstream processing (Nandakumar et al. 2006). This was the case of Bliβ expression, and therefore we tested the application of its biosynthetic pathway to other peptides, including non-lanthipeptides. The results showed that the outcome depended greatly on the peptide tested. Forty-three percent of them (insulin A, amylin (non-lanthipeptides), and epidermin) were successfully produced and secreted without LicA2 leader peptide. However, plantaricin E and lunasin were not secreted, although they were detected in the soluble cellular fraction. Two peptides were not detected at all (somatostatin-14 and mersacidin), suggesting they are stored in inclusion bodies or that *E. coli* is able to degrade them immediately after translation. Taken together, and although optimization is required, these results show that the fusion of the LicA2 leader sequence to other peptides may direct the proteolytic activity of LicP and LicT, as well as the secretion of the core peptides to the supernatant. The results presented here expand the knowledge on LicT and LicP flexibility, with respect to the C-terminal sequences attached to LicA2 recognition sequence that were so far limited to other lanthipeptides and their specifically designed analogues (prochlorosin, nisin, haloduracin) (Tang et al. 2015; Si et al. 2018). The expression systems of these fusions will benefit from further optimization, but they show that it is possible to expand the use of lanthipeptide’s biosynthetic enzymes to other biotechnological applications, contributing to the increased application of microorganisms as biofactories.

## Supplementary Information

Below is the link to the electronic supplementary material.

Supplementary file1. The supporting information is available free of charge. Supplemental figures and tables, including: a standard calibration curve, lists of primers and molecule masses as well as MS spectra (PDF) (PDF 1609 KB)

## Data Availability

All data obtained in this study is provided either within the manuscript or the corresponding supplementary material.
