# Supplementary material for: Class II two-peptide lanthipeptide proteases: exploring LicTP for biotechnological applications
Source: Appl Microbiol Biotechnol. 2023 Feb 10;107(5-6):1687–96. doi: 10.1007/s00253-023-12388-5 (PMC10006061; doi:10.1007/s00253-023-12388-5)
Supplement: Supplementary file 1 — Supplementary file1. The supporting information is available free of charge. Supplemental figures and tables, including: a standard calibration curve, lists of primers and molecule masses as well as MS spectra (PDF) (PDF 1609 KB) [file 253_2023_12388_MOESM1_ESM.pdf]

Supplementary Information

Class II two-peptide lanthipeptides proteases: exploring LicTP for biotechnological applications

Joana C. Barbosa[1], Tânia Caetano[1], Eva Mösker[2], Roderich D. Süssmuth[2], Sónia Mendo[1]

[1] Department of Biology & Centre for Environmental and Marine Studies (CESAM), University of Aveiro, Aveiro, Portugal

[2] Institut für Chemie, Technische Universität Berlin, Berlin, Germany

* Corresponding author: Sónia Mendo; Departamento de Biologia, Universidade de Aveiro, Campus Universitário de Santiago, 3810-193 Aveiro, Portugal; smendo@ua.pt

Supplementary Figures

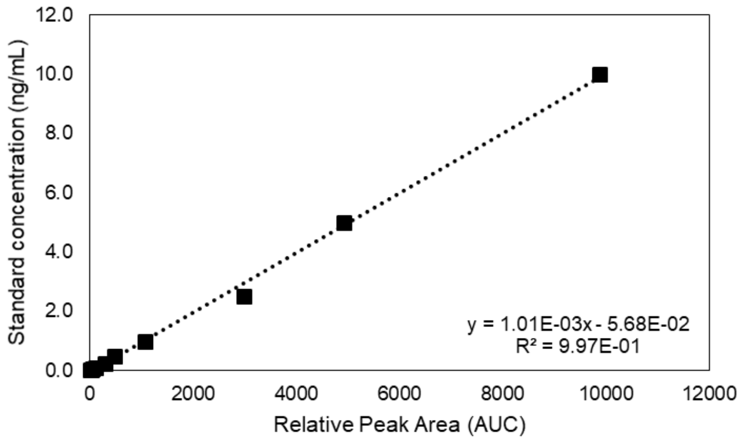

Figure S1. Lichenicidin standard calibration curve for Bliβ. Liquid stocks of purified Bliβ peptide were prepared in 70% ACN. Concentrations applied for MS measurements were 0.01, 0.025, 0.05, 0.1, 0.25 0.5, 1, 2.5, 5 and 10 ng/mL. The concentration was plotted against the resulting peak area in MRM measurements. Best fit line and coefficient of determination R^2 were calculated as indicated.

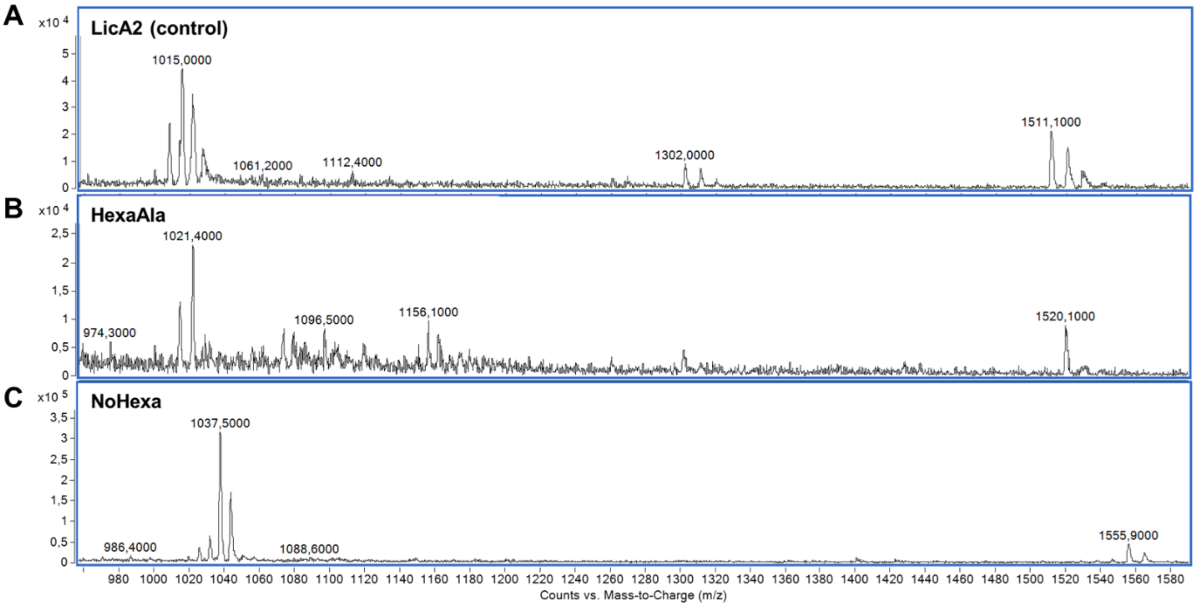

D

	Expected mass	$M+H^{2+}$	$M+H^{2+}$	Dehydrations
LicA2 (expected)	3019	1510	1007	12
HexaAla	3037	1519	1013	11 (+1 H_2O)
NoHexa	3109	1555	1037	7 (+5 H_2O)

Figure S2. MS Scan spectra of the hexapeptide variants extracts. Spectrum of extracts containing the control LicA2 (A), HexaAla (B) and NoHexa (C) variants. (D) masses obtained for each variant and the predicted changes, in terms of dehydrations, in comparison with the control.

Table S1. List of primers, and respective annealing temperatures, used in the SDM of Bliβ hexapeptide and GG-motif. Primers were designed using PrimerX to generate lichenicidin variants. The mutated codons are underlined. (^) indicates a deletion position.

Mutation	Primer sequence (5' → 3')	Annealing temperature (ºC)
Glu-1Ala	Fw: TGACGTCAATCCTGCGACAACTCCTGCTA<br>Rv: TAGCAGGAGTTGTCGCAGGATTGACGTCA	55
Glu-1Asp	Fw: GTCAATCCTGATACAACTCCTGC<br>Rv: GCAGGAGTTGTATCAGGATTGAC	50
Glu-1Gln	Fw: CGTCAATCCTCAGACAACTCCTG<br>Rv: CAGGAGTTGTCTGAGGATTGACG	52
Pro-2Ala	Fw: AAATGACGTCAATGCGGAAACAACTCCTG<br>Rv: CAGGAGTTGTTTCCGCATTGACGTCATTT	55
Asn-3Ala	Fw: AGGAAATGACGTCGCGCCTGAAACAACTCC<br>Rv: GGAGTTGTTTCAGGCGCGACGTCATTTCCT	55
Asn-3His	Fw: AATGACGTCCATCCTGAAAC<br>Rv: GTTTCAGGATGGACGTCATT	48
Asn-3Gln	Fw: GAAATGACGTCCAGCCTGAAACAAC<br>Rv: GTTGTTTCAGGCTGGACGTCATTTC	54
Val-4Ala	Fw: AGGAGGAAATGACGCGAATCCTGAAACAA<br>Rv: TTGTTTCAGGATTCGCGTCATTTCCTCCT	55
Asp-5Ala	Fw: GGTAGGAGGAAATGCCGTCAATCCTGAAAC<br>Rv: GTTTCAGGATTGACGGCATTTCCTCCTACC	55
Asn-6Ala	Fw: CTTTGGTAGGAGGAGCGGACGTCAATCCTG<br>Rv: CAGGATTGACGTCCGCTCCTCCTACCAAAG	55
Asn-6Gly	Fw: GGTAGGAGGAGGCGACGTCAAT<br>Rv: ATTGACGTCGCCTCCTCCTACC	56
Asn-6Ser	Fw: GTAGGAGGAAGCGACGTCAAT<br>Rv: ATTGACGTCGCTTCCTCCTAC	52
no_hexa	Fw: TGAAAGCTTTGGTAGGAGGAACAACTCCTGCTACAAC<br>Rv: GTTGTAGCAGGAGTTGTTCCTCCTACCAAAGCTTTCA	55
HexaAla (two sequential PCR reactions)	Fw1: CTTTGGTAGGAGGAGCAGCAGCCAATCCTGAAACAAC<br>Rv1: GTTGTTTCAGGATTGGCTGCTGCTCCTCCTACCAAAG<br>Fw2: GTAGGAGGAGCAGCAGCCGCAGCAGCAACAACTCCTGCTACAAC<br>Rv2: GTTGTAGCAGGAGTTGTTGCTGCTGCGGCTGCTGCTCCTCCTAC	55
2*hexa	Fw: CGTCAATCCTGAAAACGACGTAAACCCAGAAACAACTCCTGCTAC<br>Rv: GTAGCAGGAGTTGTTTCTGGGTTTACGTCGTTTTCAGGATTGACG	55
5Ala_Glu-1	Fw: GCCGCTGCTGAAACAACTCC<br>Rv: GGAGTTGTTTCAGCAGCGGC	62
GG-AA	Fw: GGAATTGAAAGCTTTGGTAGCAGCAAATGACGTCAATCCTGAAA<br>Rv: TTTCAGGATTGACGTCATTTGCTGCTACCAAAGCTTTCAATTCC	55
GG-GA	Fw: TTTGGTAGGAGCGAATGACGTCA<br>Rv: TGACGTCATTCGCTCCTACCAAA	53
GG-GS	Fw: CTTTGGTAGGATCAAATGACGTC<br>Rv: GACGTCATTTGATCCTACCAAAG	50

Table S2. List of primers used for amplification of the chimeric genes. The annealing temperatures, size of the amplicons and restriction sites (**bold**) of each primer are also indicated. In the last column the restriction enzymes employed in each case is indicated; when shown between brackets, the restriction site is encoded within the amplicon and not in the primer itself.

**Amplicon**	**Primer sequence (5’ → 3’)**	**Annealing temperature (ºC)**	**Size (bp)**	**Restriction enzyme**
licT	Fw: TAG**CCATGG**CAGGAGGTATAAGGCATGTTTTTTCATAAGA<br>Rv: GGTGGTGGTG**GTCGAC**TCACATCATCACCTCTGCAGATT	58	2157	SalI<br>NcoI
licP	Fw: TATA**CCATGG**AAAAGAATATATATTTTTCTCT<br>Rv: TTTTAT**GTCGAC**TCACTCCTTGTTCATCATTTT	50	1334	SalI<br>NcoI
licT.P	Fw: AGGTGA**CCATGG**GCTTGTTTTTTCATAAGACACCG<br>Rv: TACA**GTCGAC**TCACTCCTTGTTCATCATTTTC	51	3623	SalI<br>NcoI
Amy	Fw: AGGGTTTTCCCAGTCACGAC<br>Rv: CATATGCTCGAGATAGGTATT	48	231	(NdeI)<br>(XhoI)
Luna	Fw: CAGGAAACAGCTATGAC<br>Rv: CAGGAAACAGCTATGAC	45	249	(NdeI)<br>(XhoI)
InsA	Fw: ACCAGCTGCCTCGAGCATATG<br>Rv: CAGGAAACAGCTATGAC	50	183	(NdeI)<br>(XhoI)
Soma	Fw: AGCATTCGCCTCGAGCATATG<br>Rv: CATATGCTCGAGGCAGCTGGT	58	162	(NdeI)<br>(XhoI)
PlnE	Fw: AGGGTTTTCCCAGTCACGAC<br>Rv: CATATGCTCGAGGCGAATGCT	53	219	(NdeI)<br>(XhoI)
Epi	Fw: CATATGAAAACCATGAAAAA<br>Rv: TGCTCGAGACAACAATAACT	50	198	(NdeI)<br>(XhoI)
Mrs	Fw: AACAGGTAGTTTTAACAGTTATTGTT<br>Rv: CTCGAGACAAATACATTCAG	50	192	(NdeI)<br>(XhoI)

Table S3. Predicted peptide molecular masses of the chimeric genes obtained in this study. “n” indicates the charge state of the peptide. The observed mass peaks are also indicated in **bold** to facilitate identification of the ionization state. The extracts were run in an ESI-qTOF mass spectrometer and the masses, except for the monoisotopic, correspond to the H+ adducts.

Amylin			
N	A2_Leader+Amylin	A2_hexa+Amylin	Amylin
Monoisotopic mass	9278.43145	5737.66755	5069.39099
1	9279.439275	5738.675375	5070.398815
2	4640.22355	2869.8416	2535.70332
3	3093.818308	1913.563675	1690.804822
4	2320.615688	1435.424713	1268.355573
5	1856.694115	1148.541335	**1014.886023**
6	1547.413067	957.28575	845.9063233
7	**1326.498032**	820.6746179	**725.2065379**
8	**1160.811756**	718.2162688	634.6816988
9	**1031.944653**	638.5264417	564.2734906
10	928.85097	574.77458	507.946924
11	844.5015932		
12	**774.2104458**		
13	714.7333212		
14	663.7529286		
15	619.5699217		
16	580.9097906		
17	546.7979103		
18	516.4762389		
19	489.3463224		
20	464.9293975		
Lunasin			
N	A2_Leader+Lunasin	A2_hexa+Lunasin	Lunasin
Monoisotopic mass	10341.79392	6801.03002	6132.75346
1	10342.80175	6802.037845	6133.761285
2	5171.904785	3401.522835	3067.384555
3	3448.272465	2268.017832	2045.258978
4	2586.456305	1701.26533	1534.19619
5	2069.366609	1361.213829	1227.558517
6	1724.640145	**1134.512828**	**1023.133402**
7	1478.406956	972.5835421	877.1154621
8	1293.732065	851.1365775	767.6020075
9	**1150.096038**	756.6778272	682.4248761
10	**1035.187217**	681.110827	614.283171
11	**941.1709086**		
12	**862.823985**		
13	**796.5304342**		
14	**739.7073907**		
15	**690.460753**		
16	647.369945		
17	609.3486438		
18	575.5519317		
19	545.3127682		
20	518.097521		
Plantaricin E			
N	A2_Leader+PlnE	A2_hexa+PlnE	PlnE

Monoisotopic mass	8917.53804	5376.77414	4708.49758
1	8918.545865	5377.781965	4709.505405
2	4459.776845	2689.394895	2355.256615
3	2973.520505	1793.265872	1570.507018
4	2230.392335	1345.20136	**1178.13222**
5	1784.515433	**1076.362653**	**942.707341**
6	1487.264165	**897.1368483**	**785.7574217**
7	1274.941831	**769.1184164**	**673.6503364**
8	1115.70008	**673.1045925**	**589.5700225**
9	991.845385	**598.4271739**	**524.1742228**
10	**892.761629**	538.685239	**471.857583**
11	**811.6931014**		
12	**744.135995**		
13	**686.9722896**		
14	**637.9748279**		
15	**595.510361**		
16	558.3539525		
17	525.5688862		
18	496.426605		
19	470.3519324		
20	446.884727		
Somatostatin			
N	A2_Leader+Soma	A2_hexa+Soma	Soma
Monoisotopic mass	7013.30054	3472.53664	2804.26008
1	7014.308365	3473.544465	2805.267905
2	3507.658095	1737.276145	1403.137865
3	2338.774672	1158.520038	935.761185
4	1754.33296	869.141985	702.072845
5	1403.667933	695.515153	561.859841
6	1169.891248	579.7639317	468.384505
7	1002.907902	497.0844879	401.6164079
8	877.6703925	435.074905	351.540335
9	780.2634406	386.8452294	312.5922783
10	702.337879	348.261489	281.433833
11	638.5806014		
12	585.4495367		
13	540.4924819		
14	501.9578636		
15	468.5611943		
16	439.3391088		
17	413.5549156		
18	390.6356328		
19	370.1289061		
20	351.672852		
Insulin A			
N	A2_Leader+InsA	A2_hexa+InsA	InsA
Monoisotopic mass	7756.56828	4215.80438	3547.52782
1	7757.576105	4216.812205	3548.535645
2	3879.291965	2108.910015	1774.771735
3	2586.530585	**1406.275952**	**1183.517098**
4	1940.149895	**1054.95892**	**887.88978**
5	1552.321481	**844.168701**	**710.513389**
6	1293.769205	**703.6418883**	**592.2624617**
7	**1109.089008**	603.2655936	507.7975136
8	970.57886	527.9833725	444.4488025

9	862.848745	469.4305339	395.1775828
10	776.664653	422.588263	355.760607
11	706.1503959		
12	647.388515		
13	597.6669235		
14	555.0484164		
15	518.112377		
16	485.7933425		
17	457.2765474		
18	431.928285		
19	409.2482608		
20	388.836239		
Epidermin			
N	A2_Leader+Epi	A2_hexa+Epi	Epi
Monoisotopic mass	7674.57804	4133.81414	3465.53758
1	7675.585865	4134.821965	3466.545405
2	3838.296845	2067.914895	1733.776615
3	2559.200505	**1378.945872**	1156.187018
4	1919.652335	**1034.46136**	**867.39222**
5	1535.923433	**827.770653**	**694.115341**
6	**1280.104165**	**689.9768483**	**578.5974217**
7	**1097.376116**	**591.5527021**	496.0846221
8	**960.33008**	517.7345925	**434.2000225**
9	**853.7387183**	460.3205072	386.0675561
10	**768.465629**	414.389239	347.561583
11	**698.6967377**		
12	**640.555995**		
13	591.3599819		
14	549.1919707		
15	512.646361		
16	480.6689525		
17	452.4535921		
18	427.3732717		
19	404.932985		
20	384.736727		
Mersacidin			
N	A2_Leader+Mrs	A2_hexa+Mrs	Mrs
Monoisotopic mass	7335.40851	3794.64461	3126.36805
1	7336.416335	3795.652435	3127.375875
2	3668.71208	1898.33013	1564.19185
3	2446.143995	1265.889362	1043.130508
4	1834.859953	949.6689775	782.5998375
5	1468.089527	759.936747	626.281435
6	1223.57591	633.4485933	522.0691667
7	1048.923326	543.0999121	447.6318321
8	917.9338888	475.3384013	391.8038313
9	816.053215	422.6350039	348.3820528
10	734.548676	380.472286	313.64463
11	667.8631441		
12	612.2918675		
13	565.2700181		
14	524.9655757		
15	490.035059		
16	459.4708569		
17	432.5024432		
18	408.53052		

19	387.0819571	
20	367.7782505	

Table S4. MS scan spectra of each of extracts from the recombinant peptides. The molecular mass signals corresponding to the individual peptides that were detected are highlighted. Where MS spectra are not present, the respective variants could not be detected.

<table>
<tr><td colspan="3">Amylin</td></tr>
<tr><td>A2_leader+Amy</td><td>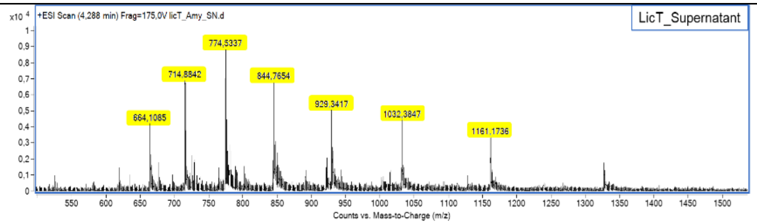

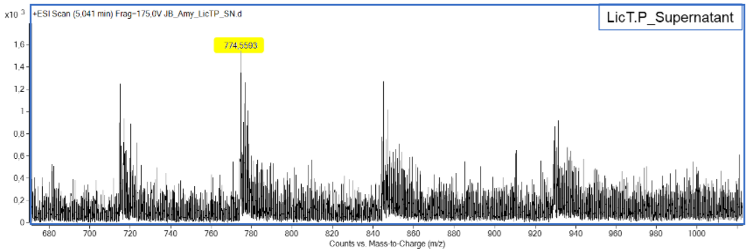
</td><td>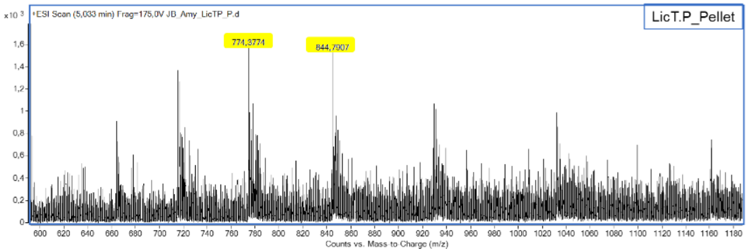
</td></tr>
<tr><td>Hexa+Amy</td><td></td><td></td></tr>
<tr><td>Amylin</td><td>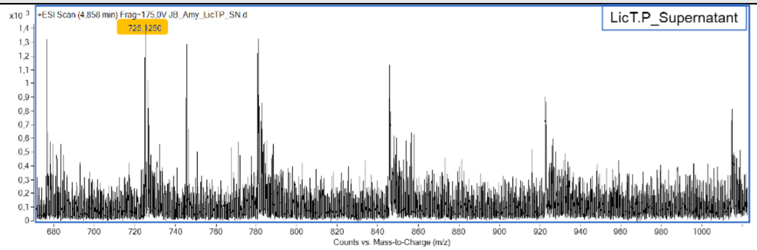
</td><td>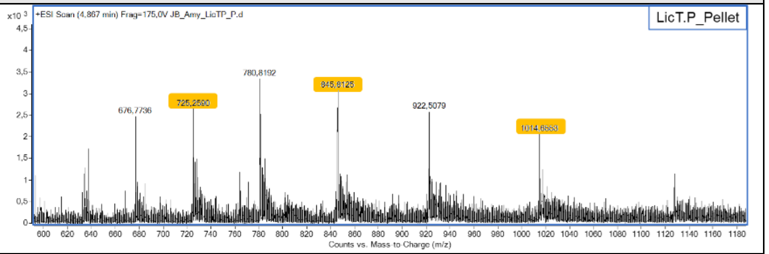
</td></tr>
</table>

<table>
<tr><th colspan="3">Lunasin</th></tr>
<tr><td>A2_leader+Luna</td><td>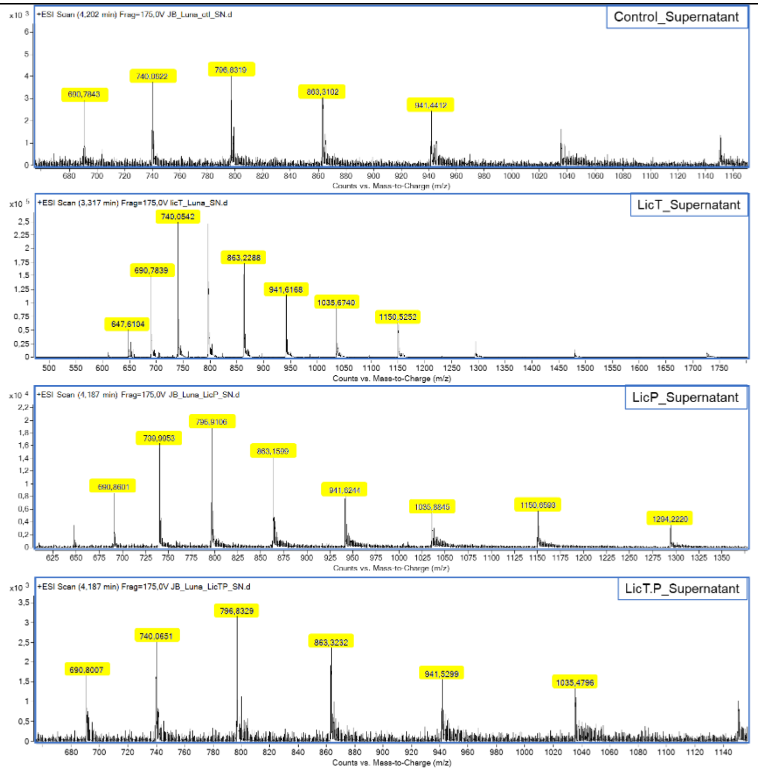
</td><td>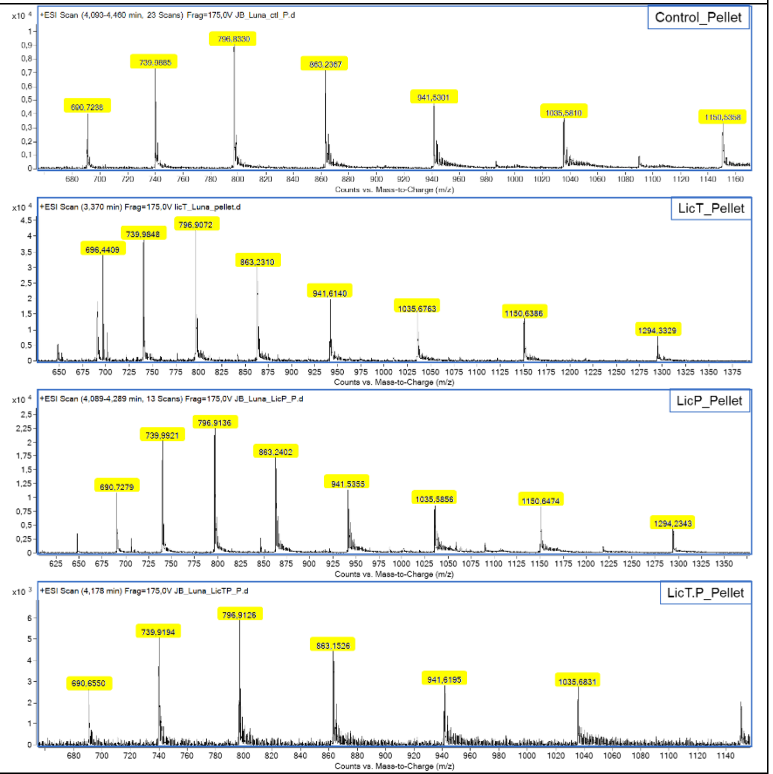
</td></tr>
<tr><td>Hexa+Luna</td><td></td><td></td></tr>
<tr><td>Lunasin</td><td></td><td></td></tr>
</table>

Insulin A

<table>
<tr>
<td>A2_leader+InsA</td>
<td>
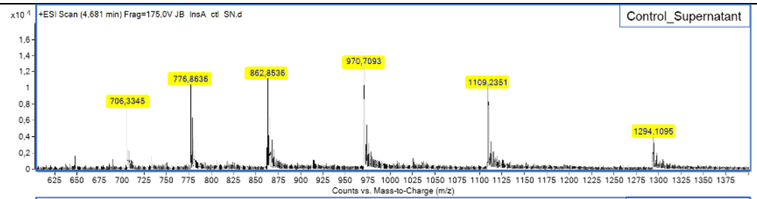

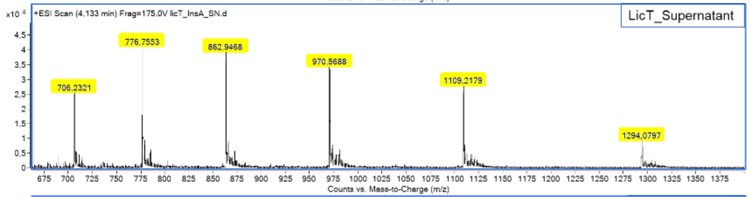

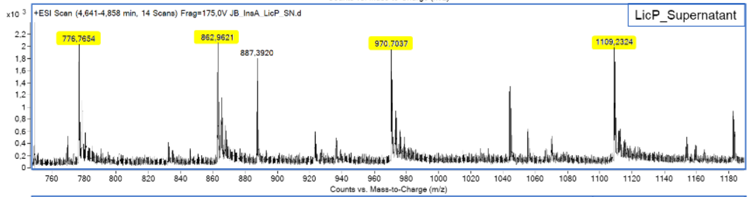

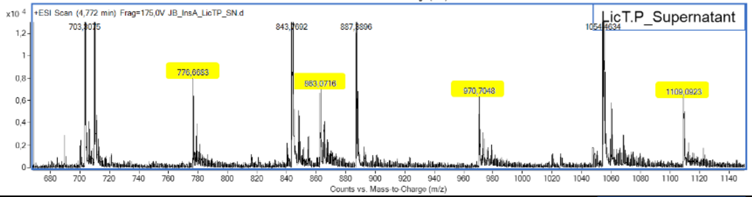

</td>
<td>
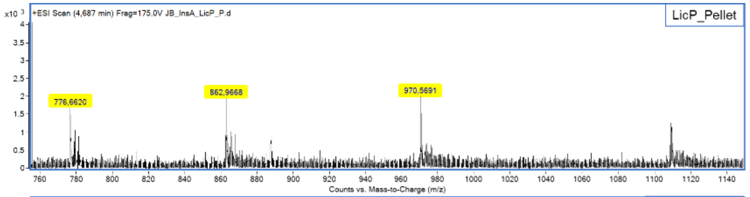

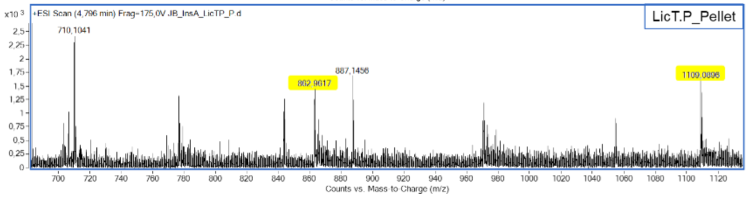

</td>
</tr>
<tr>
<td>Hexa+InsA</td>
<td>
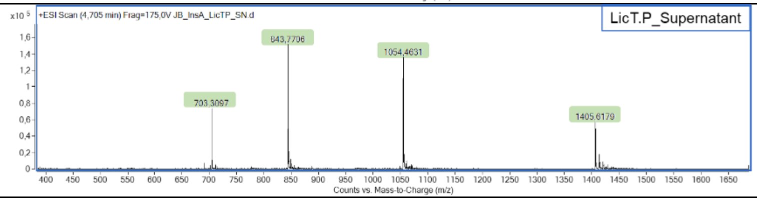

</td>
<td>
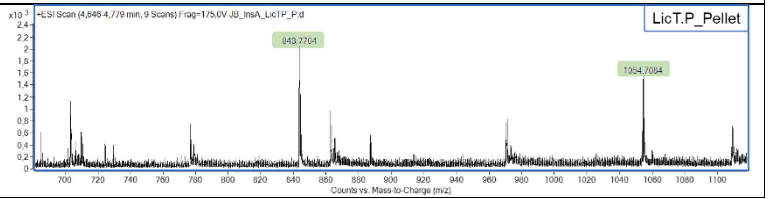

</td>
</tr>
</table>

Insulin A	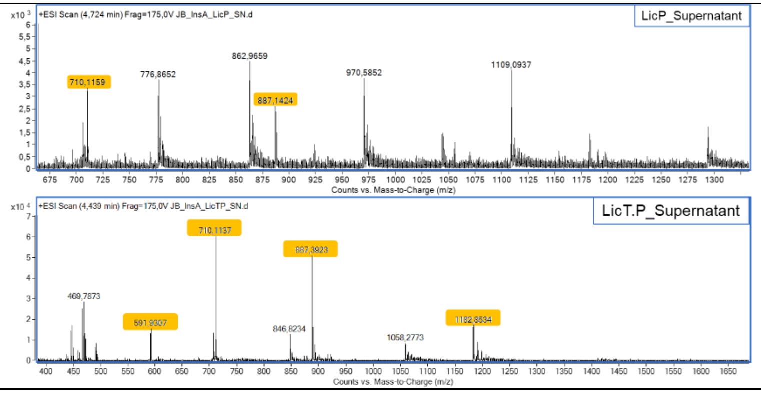	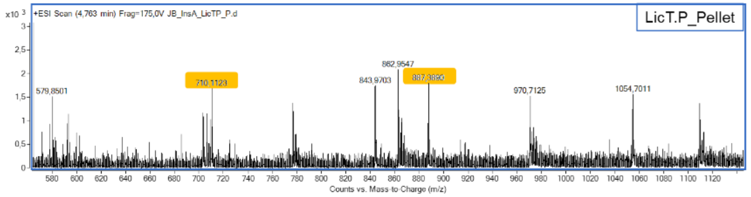

LicP_Supernatant
+ESI Scan (4,724 min) Frag=175,0V JB_InsA_LicP_SN.d
710,1159
776,8652
862,9659
887,1424
970,5852
1109,0937
Counts vs. Mass-to-Charge (m/z)
LicT.P_Supernatant
+ESI Scan (4,439 min) Frag=175,0V JB_InsA_LicTP_SN.d
469,7673
591,9507
710,1137
846,8234
887,3923
1058,2773
1182,8634
Counts vs. Mass-to-Charge (m/z)

LicT.P_Pellet
+ESI Scan (4,763 min) Frag=175,0V JB_InsA_LicTP_P.d
579,8501
710,1123
843,9703
862,9547
887,3890
970,7125
1054,7011
Counts vs. Mass-to-Charge (m/z)

**Plantaricin E**			
A2_leader + PlnE	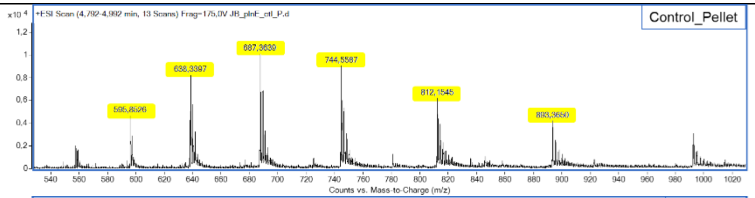<br>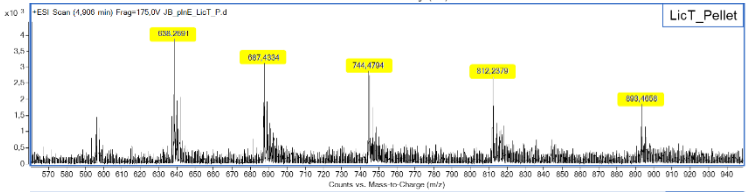<br>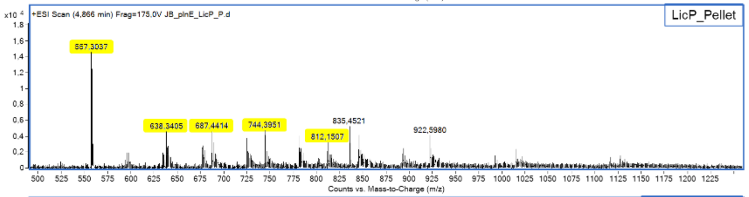<br>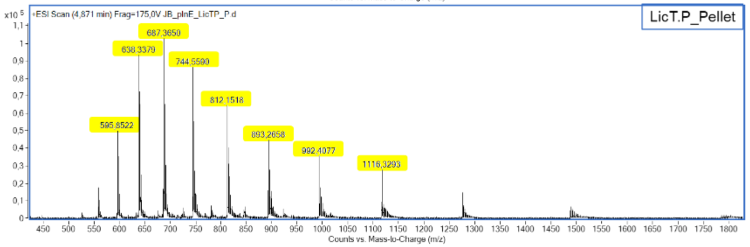	Hexa + PlnE	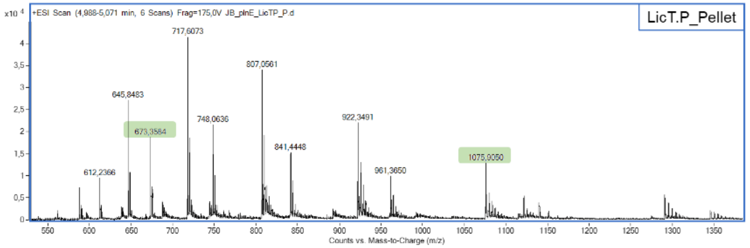
		Pln E	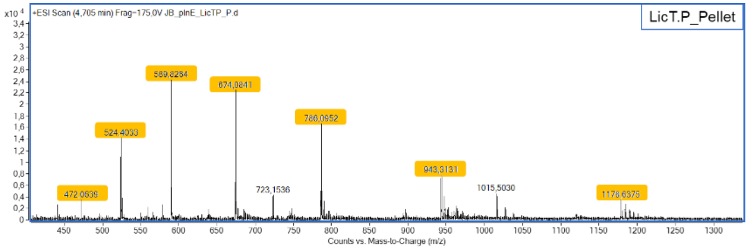

**Epidermin**		
A2_leader+Epi	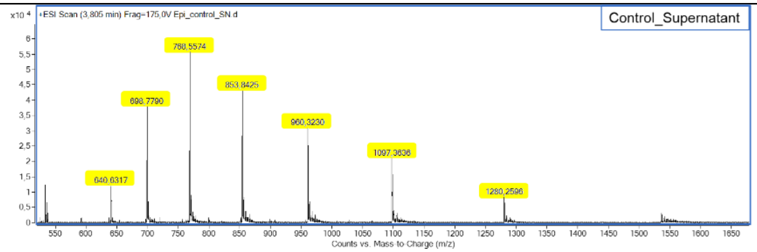<br>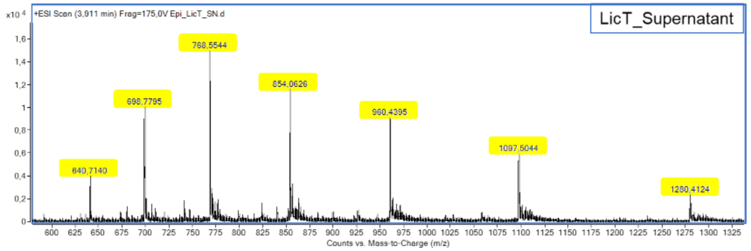<br>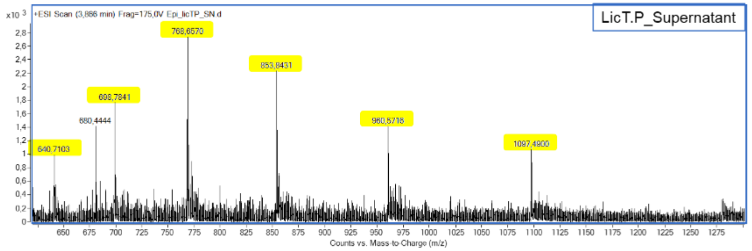	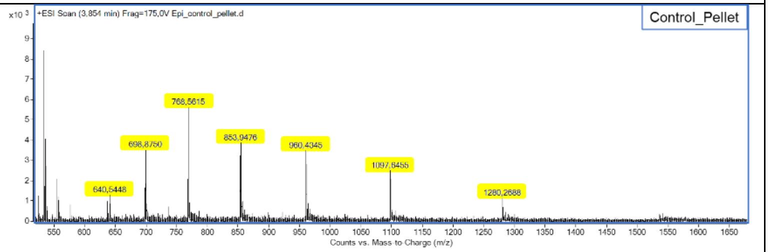<br>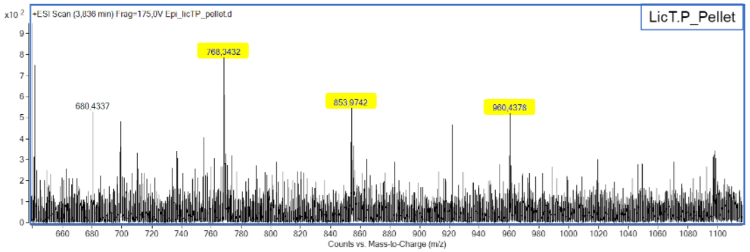
Hexa+Epi	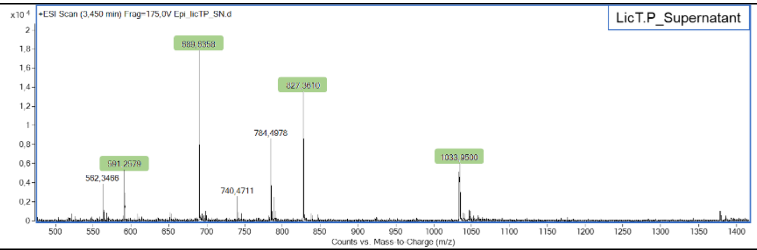	

Epidermin	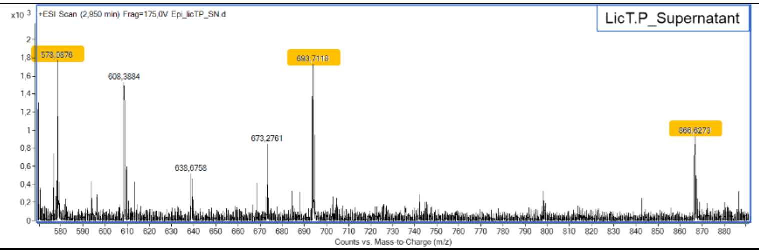x10 3<br>+ESI Scan (2,950 min) Frag=175,0V Epi_licTP_SN.d<br>LicT.P_Supernatant<br>578,0876<br>608,3884<br>638,6758<br>673,2761<br>693,7118<br>866,6273<br>0 0,2 0,4 0,6 0,8 1 1,2 1,4 1,6 1,8 2<br>580 590 600 610 620 630 640 650 660 670 680 690 700 710 720 730 740 750 760 770 780 790 800 810 820 830 840 850 860 870 880<br>Counts vs. Mass-to-Charge (m/z)	
